# Centrifugation Conditions in the L-PRP Preparation Affect Soluble Factors Release and Mesenchymal Stem Cell Proliferation in Fibrin Nanofibers

**DOI:** 10.3390/molecules24152729

**Published:** 2019-07-27

**Authors:** Bruna Alice Gomes de Melo, Ângela Cristina Malheiros Luzo, José Fabio Santos Duarte Lana, Maria Helena Andrade Santana

**Affiliations:** 1 Department of Engineering of Materials and Bioprocesses, School of Chemical Engineering, University of Campinas, Campinas, SP 13083-852, Brazil; 2 Hematology & Hemotherapy Center, Umbilical Cord Blood Bank, University of Campinas, Campinas, SP 13083-878, Brazil; 3 Bone and Cartilage Institute, Indaiatuba, SP 13334-170, Brazil

**Keywords:** platelet, leukocyte, L-PRP, centrifugation, fibrin, nanofiber, growth factor, cytokine, mesenchymal stem cells

## Abstract

Leukocyte and platelet-rich plasma (L-PRP) is an autologous product that when activated forms fibrin nanofibers, which are useful in regenerative medicine. As an important part of the preparation of L-PRP, the centrifugation parameters may affect the release of soluble factors that modulate the behavior of the cells in the nanofibers. In this study, we evaluated the influences of four different centrifugation conditions on the concentration of platelets and leukocytes in L-PRP and on the anabolic/catabolic balance of the nanofiber microenvironment. Human adipose-derived mesenchymal stem cells (h-AdMSCs) were seeded in the nanofibers, and their viability and growth were evaluated. L-PRPs prepared at 100× *g* and 100 + 400× *g* released higher levels of transforming growth factor (TGF)-β1 and platelet-derived growth factor (PDGF)-BB due to the increased platelet concentration, while inflammatory cytokines interleukin (IL)-8 and tumor necrosis factor (TNF)-α were more significantly released from L-PRPs prepared via two centrifugation steps (100 + 400× *g* and 800 + 400× *g*) due to the increased concentration of leukocytes. Our results showed that with the exception of nanofibers formed from L-PRP prepared at 800 + 400× *g*, all other microenvironments were favorable for h-AdMSC proliferation. Here, we present a reproducible protocol for the standardization of L-PRP and fibrin nanofibers useful in clinical practices with known platelet/leukocyte ratios and in vitro evaluations that may predict in vivo results.

## 1. Introduction

In the past few years, the benefits of autologous leukocyte- and platelet-rich plasma (L-PRP) have been evidenced in the treatment of many types of diseases [1,2,3,4,5,6]. Aside from growth factors (GFs) released from the platelets’ alpha granules, L-PRP contains inflammatory cytokines secreted from leukocytes that act in synergy to modulate the migration, proliferation, and differentiation of autologous cells through different pathways that lead to tissue regeneration [7,8,9,10,11]. Depending on the site, the degree of the injury (acute or chronic), and treatment phase (early or late stage of healing), the leukocyte fraction must be adjusted from poor-leukocyte PRP (P-PRP) to L-PRP [12,13,14]. Modern classifications systems consider the platelet and leukocyte levels, aside from other conditions, such as the number of centrifugation spins, activation, the presence of erythrocytes, and guided applications [15,16,17,18].

Whether conducted manually or by machine, L-PRP is prepared by centrifuging the patient’s whole blood, in which platelets, leukocytes, proteins, and other components are concentrated in a small fraction of plasma, with their levels adjusted by varying the centrifugation conditions [19,20]. In a previous study, we determined the distribution and recovery of platelets and leukocytes (lymphocytes and granulocytes) in the supernatant, buffy coat, and erythrocyte layers by centrifuging the whole blood from 100 to 800× *g* (10 min and 25 °C). The concentration patterns allowed for the identification of specific centrifugation ranges to obtain distinct platelet/leukocyte and the associate lymphocyte/granulocyte ratios. As blood centrifugation is a separation process based on the size and density of the components as well as the packing behavior of the erythrocytes, different recoveries of platelets and leukocytes could be obtained in L-PRP from a first spin [19]. In turn, a second centrifugation step under accelerations higher than 400× *g* could lead to platelet aggregation and deactivation [20,21]. Therefore, choosing the centrifugation conditions for L-PRP preparation is crucial to achieving an optimized performance.

When activated, L-PRP forms a 3D network composed of fibrin nanofibers that act as a scaffold for autologous cells to migrate, proliferate, and differentiate at the injury site. Various works in the literature have used fibrin nanofibers as a scaffold for tissue engineering, whether obtained from commercial fibrinogen [22,23,24] or from PRP formulations [25,26,27], and have reported on their suitability in regenerative medicine. However, fibrin from PRP presents advantages over commercial fibrin due to its rich microenvironment, with many GFs and cytokines being gradually released from the nanofibers, thus stimulating the regenerative process [28,29]. The PRP type and preparation conditions directly influence the final architecture of the fibrin networks in terms of the nanofiber thickness, length, and organization [30]. While P-PRP and L-PRP favor the formation of low-density fibrin, P-PRF and L-PRF (pure platelet-rich fibrin and leukocyte- and platelet-rich fibrin) favor the formation of high-density fibrin [16], which could affect the GFs and the entrapment and release dynamics of the cytokines [28].

Transforming GF (TGF-β) and platelet-derived GF (PDGF) are the most abundant GFs present in the platelets’ alpha granules and are primarily responsible for modulating the wound healing process [31]. They act in the early stages of healing by creating a concentration gradient to attract inflammatory agents, macrophages, mesenchymal stem cells (MSCs), fibroblasts, and other cells to the injury site, and also stimulate the secretion of more GFs, cell proliferation, differentiation, and the production of extracellular matrix (ECM) [11,32,33].

Leukocytes are known to secrete inflammatory cytokines, such as interleukin (IL)-8 and tumor necrosis factor (TNF)-α, which leads to ECM degradation [34,35]. However, they play essential roles in healing during the inflammatory phase, acting as immunoregulatory and antibacterial agents, as well balancing the anabolic/catabolic microenvironment [10,36,37,38]. Therefore, an optimized concentration of cytokines is crucial for contributing to the regenerative process and the maintenance of tissue homeostasis [37,39,40].

Associated with endogenous MSCs, L-PRP and fibrin nanofibers constitute the proliferation triangle, a term previously created to identify all necessary components to achieve effective tissue regeneration (3D matrix, signaling molecules, and progenitor cells) [31,41]. Due to its high availability and great chondrogenic and osteogenic capability, exogenous MSCs have been used in cell-therapies and cartilage and bone engineering studies, presenting synergistic effects with L-PRP, such as increased GF and cytokine secretion, cell migration, proliferation, and differentiation [42,43,44].

In this work, we aimed to study the effects of different centrifugation conditions on the preparation of L-PRP, the release kinetics of GFs and cytokines, and on the response of MSCs from human adipose-derived tissue (h-AdMSCs) seeded in fibrin nanofibers. The results obtained represent an advanced step toward understanding the effects of the platelet/leukocyte ratio and catabolic/anabolic balance on cell behavior. The standardized preparations reduce variability and advise physicians and researchers to select the parameters and operational conditions to obtain L-PRP and fibrin nanofibers with optimal yield and performance.

## 2. Results

**Figure 1:**
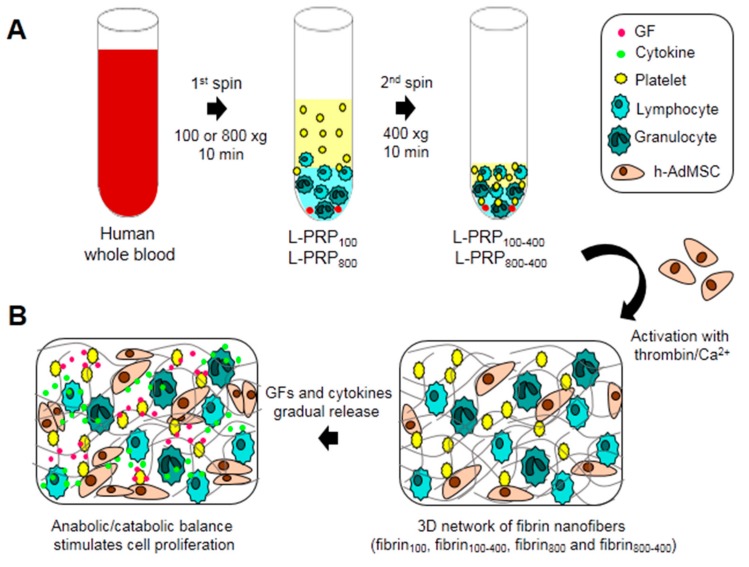
Schematic illustration of the experimental design. (**A**) Human whole blood was collected and centrifuged at 100 and 800× g for 10 min to form L-PRP_100_ and L-PRP_800_. These L-PRPs were submitted to a second spin at 400 xg for 10 min to form L-PRP_100-400_ and L-PRP_800-400_ after the removal of 70% of the supernatant; (**B**) h-AdMSCs were resuspended in each L-PRP, followed by the formation of a fibrin nanofiber network after activation with thrombin/Ca^2+^. Activated platelets and leukocytes secrete GFs and cytokines that in an appropriate balance stimulate cell proliferation.

L-PRP was prepared by centrifuging the whole blood at two accelerations, 100 and 800× g, using one or two spin steps to form four formulations: L-PRP_100_, L-PRP_100-400_, L-PRP_800_, and L-PRP_800-400_ (Figure 1A). L-PRP activation led to the formation of fibrin nanofibers (fibrin_100_, fibrin_100-400_, fibrin_800_, and fibrin_800-400_) rich in GFs and cytokines that are suitable for supporting cells, and whose response depend on the anabolic/catabolic balance of the microenvironment (Figure 1B).

**Figure 2:**
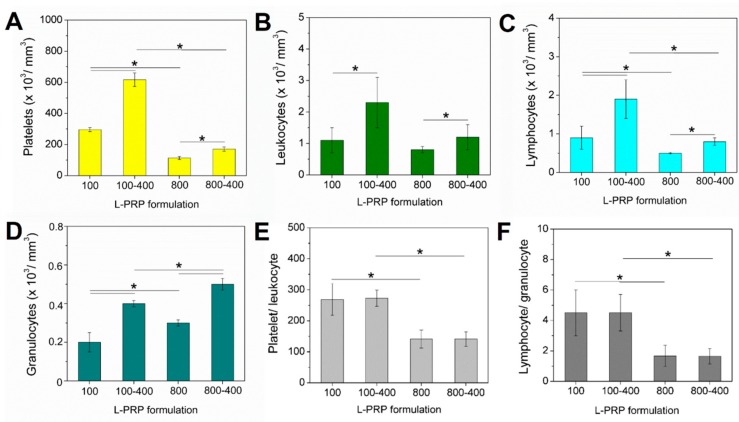
Concentration of (**A**) platelets, (**B**) leukocytes, (**C**) lymphocytes, and (**D**) granulocytes, obtained by measuring each L-PRP in a hematologic analyzer. The calculated ratio of (**E**) platelet/leukocyte and (**F**) lymphocyte/granulocyte. Note: * *p* < 0.05.

The concentrations of platelets, total leukocytes, lymphocytes, and granulocytes in the whole blood were 149 ± 33, 3.8 ± 0.9, 1.4 ± 0.9, and 2.3 ± 0.4, respectively. The slower acceleration (100× *g*) allowed the recovery of an increased concentration of platelets, especially after two centrifugation steps (Figure 2A), as also observed for the total leukocytes (Figure 2B). Lymphocytes represented the highest leukocyte portion, being highly concentrated in L-PRP_100-400_ (Figure 2C). On the other hand, the granulocyte concentration was higher in L-PRP_800_ and L-PRP_800-400_ when compared to L-PRPs from 100× *g* (Figure 2D). Therefore, the platelet/leukocyte and lymphocyte/granulocyte ratios were greater in both L-PRP_100_ and L-PRP_100-400_ in comparison to L-PRP_800_ and L-PRP_800-400_ (Figure 2E,F).

**Figure 3:**
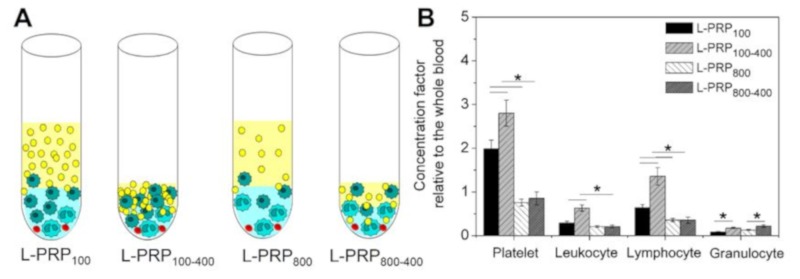
(**A**) Schematic illustration representing the concentration of the blood components in each L-PRP. (**B**) The calculated concentration factors relative to the whole blood. Note: * *p* < 0.05.

The concentration factor of all components in each L-PRP was calculated relative to the whole blood, and values varied according to the applied centrifugation condition (Figure 3A). The highest value was obtained for platelets in L-PRP_100_ and L-PRP_100-400_, which were 1.98 ± 0.2 and 2.8 ± 0.3, respectively. The high speed significantly decreased the concentration factor of the platelets, which did not reach the baseline at ~0.15 for L-PRP_800_ and L-PRP_800-400_. At this speed, the concentration factor was below 1 for these two formulations due to the low concentration of granulocytes. However, a significant increase in this component was observed after the second centrifugation, which was 0.17 ± 0.02 and 0.22 ± 0.03 for L-PRP_100-400_ and L-PRP_800-400_, respectively. Among all formulations, the highest concentration factor of lymphocytes was observed in L-PRP_100-400_ (1.35 ± 0.2) (Figure 3B).

**Figure 4:**
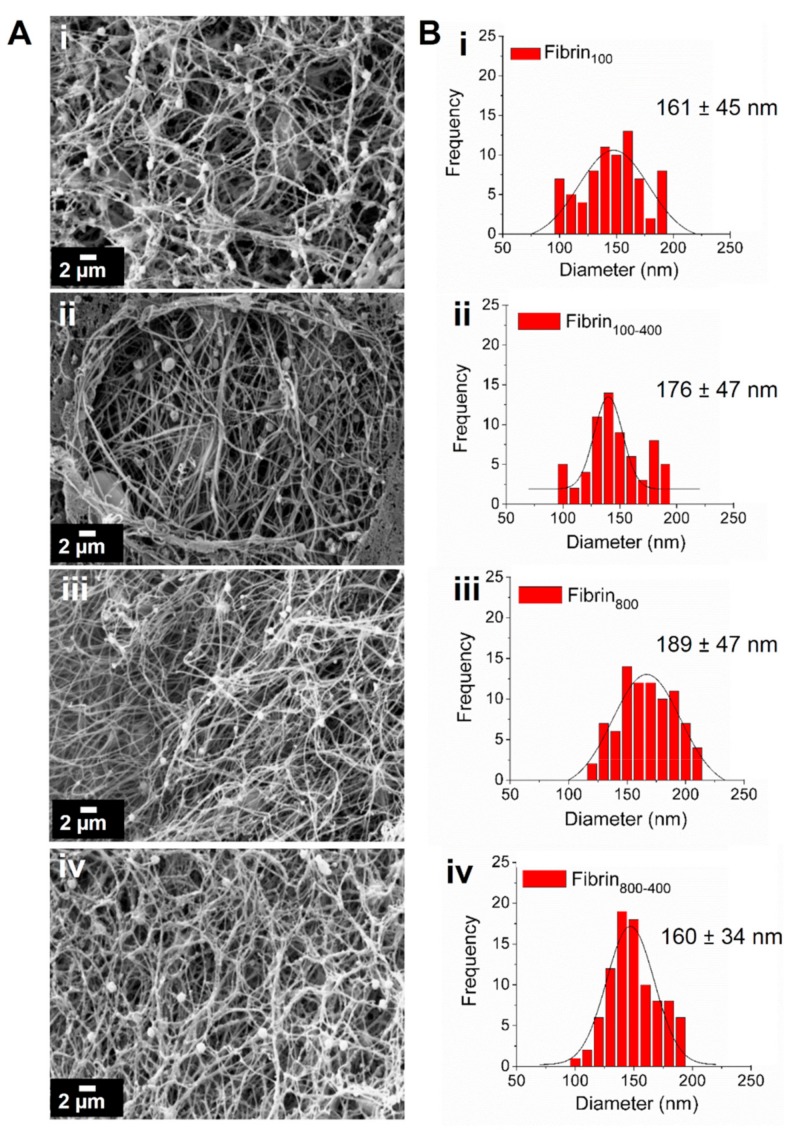
Characterization of the fibrin nanofibers. (**A**) SEM images showing the morphology of the networks. (**B**) Frequency of fiber diameter measured using ImageJ software, highlighting their mean diameter; (**i**) fibrin_100_, (**ii**) fibrin_100-400_, (**iii**) fibrin_800_, and (**iv**) fibrin_800-400_.

Fibrin nanofibers were formed after the activation of L-PRPs with thrombin/Ca^2+^. The scanning electron microscopy (SEM) images showed no substantial changes in morphology, with the structures presenting high porosity and interconnected nanofiber organization (Figure 4A). For all samples, the fiber diameters varied from 100 to 200 nm, with less polydispersity being observed for nanofibers prepared with L-PRPs via two centrifugation steps (Figure 4B).

**Figure 5:**
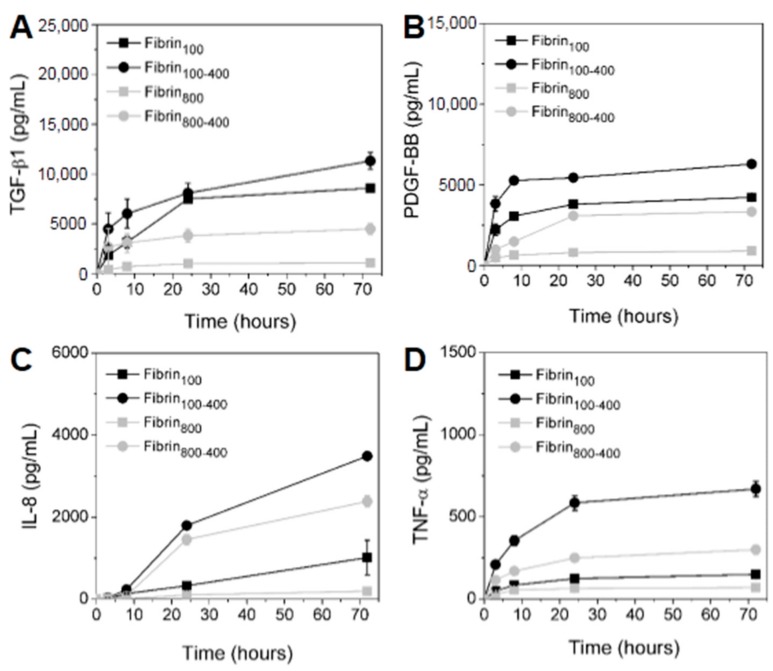
Kinetics of the GFs (**A**) TGF-β1, (**B**) PDGF-BB, and the inflammatory cytokines (**C**) IL-8 and (**D**) TNF-α released from the nanofibers over 72 h.

The cumulative concentration of GFs and cytokines released from each network was measured over 72 h (Figure 5). It was observed that GFs released from nanofibers increased proportionally with an increase in platelets in L-PRP. PDGF-BB presented a fast release in the first 3 h for fibrin_100_ and fibrin_100-400_, reaching 4230 ± 234 and 6290 ± 150 pg/mL at 72 h, respectively. For fibrin_800-400_, the release was slower, but concentration reached a high level at 72 h at 3335 ± 65 pg/mL. TGF-β1 presented the highest level among all GFs, as it was gradually released over time. Its maximum concentration was 8576 ± 305, 11,315 ± 840, 1092 ± 47, and 4490 ± 545 pg/mL for fibrin_100_, fibrin_100-400_, fibrin_800_, and fibrin_800-400_, respectively (Figure 5A). The PDGF-BB concentration released from fibrin_800_ was the smallest and reached equilibrium rapidly, with values remaining below 1000 pg/mL (Figure 5B).

The inflammatory cytokine IL-8 was highly secreted, and its concentration increased with the increase in leukocytes in L-PRP (Figure 5C). In the first 24 h, fibrin_100-400_ and fibrin_800-400_ presented a similar trend of release kinetics. Then, the IL-8 secretion sharply increased for fibrin_100-400_, reaching 3490 ± 70 pg/mL at 72 h, while for fibrin_800-400_, the value was 2382 ± 136 pg/mL. For fibrin_100_, the IL-8 kinetics was slow up to 24 h, then increased sharply after this time, reaching 1013 ± 420 pg/mL. The concentration of this cytokine in fibrin_800_ was below 200 pg/mL. TNF-α was significantly higher when released from fibrin_100-400_ in comparison to the other formulations during all of the times studied, and was ~2.5-fold and ~4-fold greater than fibrin_800-400_ and fibrin_100_, respectively (Figure 5D). Similar to IL-8, the concentration of TNF-α released from fibrin_800_ presented the smallest value (<100 pg/mL).

The L-PRP formulations were also prepared using the whole blood of two other different donors (donor 2 and donor 3), and even though they showed an initial concentration of platelets and leukocytes that were significantly different from the current donor (Figure 2 and Figure S1, Supplementary Materials), the results presented a similar trend for GF and cytokine release (Figure 5 and Figure S2, Supplementary Materials). GF concentration was proportional to the platelet/leukocyte ratio, whereas cytokines were proportional to the lymphocyte/granulocyte ratio. The lowest lymphocyte/granulocyte ratio observed for the current donor was reflected in the highest levels of TNF-α due to the predominance of granulocytes, as compared to donors 2 and 3.

**Figure 6:**
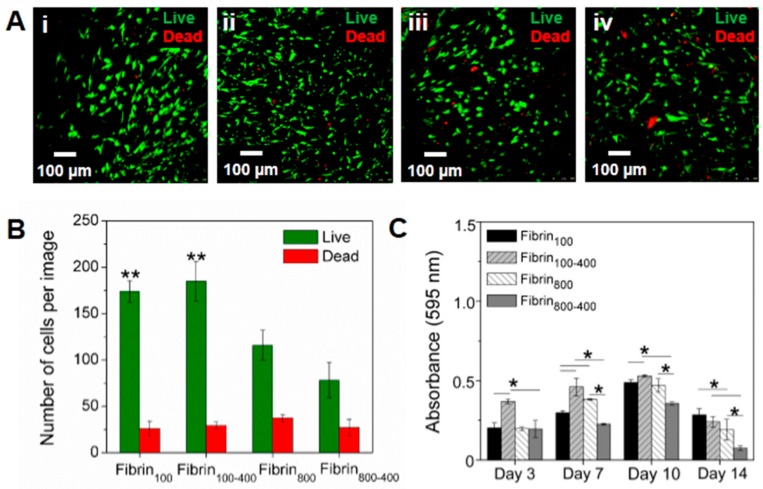
Assessment of h-AdMSC behavior seeded in the four types of fibrin nanofibers. (**A**) Cell viability on the seventh day of incubation in (i) fibrin_100_, (ii) fibrin_100-400_, (iii) fibrin_800_, and (iv) fibrin_800-400_. (**B**) Number of living and dead h-AdMSCs in the nanofibers incubated for seven days and calculated from three different images using ImageJ software. Note: ** Significantly different from fibrin_800_ and fibrin_800-400_ (*p* < 0.05). (**C**) Proliferation kinetics for h-AdMSCs seeded in the fibrin networks of the L-PRP preparations. Note: *p < 0.05.

The h-AdMSCs were cultured in the nanofibers, and their behavior in the different microenvironments was assessed as the viability and proliferation rate. Images of living and dead cells were captured in a fluorescence confocal microscope using live/dead reagents after seven days of culture. Results showed that the fibrin networks were suitable to maintain cell viability, with h-AdMSCs presenting a slightly elongated to rounded shape within the networks (Figure 6A). On day 7, the number of viable cells in the samples prepared with gentle centrifugal acceleration was significantly higher than that in the samples prepared at higher speed, with viability being 87.2 ± 2.9, 86.1 ± 2.8, 75.6 ± 0.6, and 74.3 ± 3% for fibrin_100_, fibrin_100-400_, fibrin_800_, and fibrin_800-400_, respectively (Figure 6B).

Figure 6C shows the proliferation kinetics of h-AdMSCs in terms of absorbance (595 nm) with time, which allowed for the estimation of the cell adaptation time (before the exponential phase), the range of the exponential phase, µ_max_, and t_d_ for the h-AdMSCs seeded in each preparation (Table 1). Values of estimated t_d_ were in accordance with the doubling of absorbances, approximately, as shown in Figure 6C. The highest adaption time was observed for fibrin_800-400_, while the lowest was for fibrin_100-400_. The exponential phase was approximately 4–5 days for all conditions. Although µ_max_ and t_d_ could not be estimated because it tended to be out of the studied range (<3), the values for the other conditions were similar, except for fibrin 800, where the t_d_ was slightly higher.

## 3. Discussion

In previous work, we reported on the mechanisms of platelet and leukocyte recovery in L-PRP by centrifugation, which is driven by size, density, and erythrocyte aggregation and sedimentation [19]. As predicted by theoretical calculations and experimentally confirmed, low centrifugal accelerations in the first spin (50–100× g) contribute to high yields of platelets in P-PRP (upper layer) [45]. If high spin ranges were used up until the 2010s [46,47], nowadays, we can see that low speeds for a first spin have become the consensus [21,48,49]. At the lower range of centrifugal accelerations, we have also observed the highest leukocyte recovery, with increased platelet/leukocyte and lymphocyte/granulocyte ratios in L-PRP, in comparison to accelerations ranging from 300 to 800× *g* [19]. As microenvironments with distinct blood component ratios lead to different biological responses, the standardization of centrifugation conditions and the study of cell responses to different types of L-PRPs is essential in predicting its behavior in vivo.

L-PRP activation leads to fibrin clot formation, a network composed of fibrin nanofibers that controls the release of soluble factors into the microenvironment and supports cell attachment, proliferation, differentiation, and the formation of new tissue [50]. As different centrifugal accelerations and leukocyte concentrations are related to morphological changes in the nanofibers [25,28,49], we analyzed the network’s morphology by SEM. Here, we observed that for all conditions, interconnected nanofibers were formed with a random organization, along with networks with high porosity. The different concentrations of blood components did not affect the fibrin morphology, therefore, we assumed that this would not interfere in the kinetic release of GFs and cytokines.

Gentle centrifugal acceleration is essential to maintain platelet integrity and retain their morphological properties and activation capacity, which is crucial for their gradual content release mechanism. Under high centrifugal forces, platelets are submitted to high shear stress that can induce aggregation, activation, and early GF release, impairing the proper functioning of L-PRP [20,21,49]. As previously reported, platelet activation increases with an increase in centrifugation speed, resulting in changes in their biological signature [21]. Here, we observed that the release of PDGF-BB and TGF-β1 was proportional to platelet concentration, which was higher for fibrin_100_ and fibrin_100-400_. For fibrin_800_, the concentration of both GFs was minimum. However, the first spin at 800× *g* did not damage the platelets, and after the second centrifugation at 400× *g*, an increased and gradual release of PDGF-BB and TGF-β1 was observed, indicating the maintenance of the platelets’ integrity. These GFs were selected for the experiment as they were the most abundant in the platelets, and were crucial in the anabolic pathways of the regeneration process by stimulating cell proliferation, differentiation, and the formation of extracellular matrix (ECM) [37,51].

Leukocytes secrete cytokines upon activation and strongly depend on the stimulus from the microenvironment [34,52]. Here, we observed that IL-8 and TNF-α were highly released from fibrin_100-400_ and fibrin_800-400_, with the concentration proportional to the number of leukocytes. As previously reported, granulocytes are capable of synthesizing and secreting TNF-α independent of stimulus [53], thus corroborating our results. TNF-α is immunostimulatory to lymphocytes and modulates the secretion of other cytokines, such as IL-8 [34]. It is also a potent neutrophil chemoattractant, recruiting these granulocytes to inflammatory sites for debridement and to fight infections [54].

By performing the experiment using the L-PRPs of different donors, we showed that the kinetic release of GFs and cytokines followed a trend based on the centrifugation condition applied, regardless of the initial platelet and leukocyte concentration. Therefore, it can be considered that our protocol is reproducible and suitable for application in clinical practice.

Although the presence of leukocytes, especially that of granulocytic neutrophils, could be harmful to cells due to their catabolic effects [38], by assessing the response of h-AdMSCs to the different microenvironments, we attested the role of anabolic/catabolic balance. Cell growth was modulated by the stimulus provided by the GFs, especially PDGF-BB and TGF-β1, which are important in the signaling pathways of MSC proliferation [55] and the inhibition of cytokines, with the anabolic/catabolic (GFs/cytokines) balance playing a major role in cell behavior [39]. The greatest proliferation stimulus was provided by the nanofibers fibrin_100_ and fibrin_100-400_, while inhibition was stronger for fibrin_800-400_. These results are in accordance with the kinetic release of GFs and cytokines, where fibrin_100-400_ showed a higher GF concentration and a trend for the lowest adaption time, while the high concentration of inflammatory cytokines and the lowest concentration of GFs in fibrin_800-400_ resulted in an imbalance of the anabolic/catabolic ratio, with cells presenting the highest adaptation time. As fibrin_800_ released the lowest concentration of inflammatory cytokines, even presenting lower levels of released TGF-β1 and PDGF-BB, its maximum absorbance on day 10 was slightly less than those of fibrin_100_ and fibrin_100-400_. In all fibrin networks, h-AdMSCs were in the death phase on day 14, probably due to the lack of physical space for cell duplication or nutrient depletion.

We concluded that rather than the absolute values of platelets or leukocytes, the platelet/leukocyte ratio drove cell behavior through the balance between the anabolic/catabolic effects. This balance is altered during injury and is reconstituted by L-PRP components, where the microenvironment returns to a beneficial balance and homeostasis. Therefore, it could be inferred that anabolic/catabolic balance might play a major role in the mechanisms that lead to h-AdMSC proliferation, which has a direct impact on tissue regeneration.

These results are coherent with the consensus that considers that the higher the concentration of platelets, the greater the anabolic effects [15]. However, we recommend a balanced platelet/leukocyte (>200) and the correspondent lymphocyte/granulocyte ratio (>1), which could present a higher efficiency in the applications of L-PRP and its nanofiber network in regenerative medicine.

## 4. Material and Methods

### 4.1. Whole Blood Collection

The Ethics Committee of the Medical Sciences School of the University of Campinas, Campinas, SP, Brazil (UNICAMP) (CAAE: 0972.0.146.000-11) approved the use of human blood in this study, which was collected from a healthy donor in a tube containing anticoagulant acid citrate dextrose solution A (ACD-A) (BD Vacutainer^®^, Dade City, FL, USA) prior to centrifugation.

### 4.2. L-PRP Preparation

Centrifugation of human whole blood (3.5 mL) was carried out in a ROTINA 380R centrifuge (Hettich Zentrifugen, Tuttlingen, Germany) with the tubes positioned at 45° relative to the rotor. In the first step, the whole blood was centrifuged at 100× *g* and 800× *g* for 10 min at 25 °C, followed by the collection of the upper and middle layers using an automatic pipette to obtain the L-PRP_100_ (1.6 mL) and L-PRP_800_ (2.2 mL). In the second step, L-PRP_100_ and L-PRP_800_ were centrifuged at 400× *g* for 10 min at 25 °C, and 70% of the supernatant (platelet-poor plasma, PPP) was discarded, thus obtaining L-PRP_100-400_ (0.48 mL) and L-PRP_800-400_ (0.66 mL). L-PRPs and the whole blood were taken to an ABX Micros ES 60 hematologic analyzer (Horiba ABX Diagnostics, Montpellier, France) for the measurement of blood components. The experiment was performed in triplicate (n = 3), and three measurements were taken for each sample.

### 4.3. Concentration Factor Calculation

The concentration factor of the platelets and leukocytes (including lymphocytes and granulocytes) was calculated relative to their concentration in the whole blood, according to Equation (1):

(1) $$Concentration\;facto\;=\;\frac{{\left[Platelet,\;Leukocyte,\;Lymphocyte,\;Granulocyte\right]}_{L-PRP}}{{\left[Platelet,\;Leukocyte,\;Lymphocyte,\;Granulocyte\right]}_{Whole\;blood}}$$

### 4.4. Fibrin Nanofiber Formation

The human whole blood was collected in a tube containing serum clot activator (Greiner Bio-One, Kremsmünster, Austria) and centrifuged at 2000× *g* for 15 min at 25 °C. After centrifugation, serum-containing thrombin was collected and mixed to 10% (*w*/*v*) CaCl_2_ (Sigma-Aldrich, St. Louis, MO, USA) in a 9:1 ratio (serum: Ca^2+^ *v*/*v*). This mixture was added to the L-PRP formulations at a concentration of 20% (*v*/*v*), which were left to polymerize for at least 30 min to form the fibrin_100_, fibrin_800_, fibrin_100-400_, and fibrin_800-400_ nanofibers.

### 4.5. Scanning Electron Microscopy (SEM) Analysis

After polymerization and the formation of the fibrin nanofibers, the networks were fixed in a 4% paraformaldehyde and 2.5 % glutaraldehyde solution prepared in phosphate buffer (PBS), pH 7.4, for 2 h at 4 °C. Dehydration of fixed hydrogels was carried out by dipping them in ethanol of different concentrations (50%, 70%, 95%, and 100%) at 15 min intervals. Then, the samples were dried at the critical point in a BAL-TEC critical point dryer (CPD) 030 dryer (Schalksmühle, Germany), coated with gold in a Sputter Coater POLARON, SC7620 (VG Microtech, Uckfield, England), and taken to a LEO Electron Microscopy/Oxford (Cambridge, England) for SEM images. Fiber sizes were obtained by measuring at least 100 fibers from three distinct images using the ImageJ software.

### 4.6. Kinetics of GFs and Cytokines Release

Fibrin nanofibers were prepared in a 48-well plate and incubated with low-glucose Dulbecco’s modified Eagle’s medium (DMEM) for 72 h in a humidified incubator at 37 °C and 5% CO_2_. At pre-determined times, the medium was collected and stored at −80 °C. Then, the cumulative concentration of the GFs (TGF-β1 and PDGF-BB) and cytokines (IL-8 and TNF-α,) was quantified using a Bio-plex Pro Kit in a Bio-plex 200 (Bio-Rad, Hercules, CA, USA). The experiment was performed in triplicate (*n* = 3) for each group.

### 4.7. Isolation and Culture of h-AdMSCs in Fibrin Nanofibers

The h-AdMSCs were isolated from the human subcutaneous adipose tissue of patients undergoing lipo-aspiration at the University Hospital, and cultured according to a previous protocol [56]. Cells from passage 5 to 8 were trypsinized and incorporated in each L-PRP at a concentration of 5 × 10^4^ cells/mL. Then, the L-PRPs were activated with the serum, which was composed of a Ca^2+^ mixture, and nanofibers containing h-AdMSCs were formed in a 48-well plate and cultured with 750 µL of DMEM for 2 weeks in a humidified incubator at 37 °C and 5% CO_2_, with the medium changed every three days. DMEM was not supplemented with fetal bovine serum (FBS) due to the high capacity of L-PRP to provide nutrients for cell survival and growth [57]. The experiment was performed in triplicate (*n* = 3) for each group.

### 4.8. Assessment of h-AdMSCs Survival and Growth

The number of viable cells in the fibrin nanofibers was assessed on the seventh day of culture using a Live/Dead Cell Imaging Kit (Thermo Fisher Scientific, Waltham, MA, USA). Samples were washed with PBS (pH 7.4), incubated with the live/dead reagent for 30 min, and taken to a confocal microscope (Leica Microscope TCS SP5 II, Wetzlar, Germany) for imaging. The numbers of living and dead cells were counted from three different images using ImageJ software. To assess the h-AdMSC proliferation rate, at 3, 7, 10, and 14 days, fibrin nanofibers containing the cells were incubated with 1 mg/mL dimethyl-thia-zol-2-yl]-2,5-diphenyltetrazolium bromide (MTT) (Sigma-Aldrich, St Louis, MO, USA) for 4 h at 37 °C. Then, MTT was replaced with dimethyl sulfoxide (DMSO), the samples were left in a shaker for 30 min, and the absorbance was measured at 595 nm. In order to calculate the maximum specific rate of cell growth (µ_max_) and cell doubling time (t_d_), a polynomial curve was adjusted to the experimental absorbance data obtained on days 3, 7, and 10. A second-degree polynomial curve adjusted the experimental data with a correlation coefficient of 1. From the polynomial equations, we determined values at small intervals within the studied range and delineated the cell exponential phase. The µ_max_ was calculated using Equation (2), considering the Monod-type kinetic for MSCs [58], and the t_d_ was calculated using Equation (3). The experiment was performed in triplicate (*n* = 3) for each group.

(2) $$\ln[{\left(abs\right)}_{t}/{\left(abs\right)}_{0}]=\mu_{max}.t$$

(3) $$t_{d}=\;\frac{\ln2}{\mu_{max}}$$

### 4.9. Statistical Analysis

The results are presented here as the mean ± standard deviation. One-way analysis of variance (ANOVA) with Tukey’s test was used with a 95% confidence level (*p* < 0.05).

## Figures and Tables

**Table 1:** Kinetic parameters for h-AdMSC proliferation seeded in the fibrin networks of the L-PRP preparations.

Condition	Adaptation Time (Day)	Range of Exponential Phase (Day)	µ_max_ * (day^−1^)	t_d_ ** (day)
**Fibrin_100_**	~4	4–9	0.15	4.53
**Fibrin_100-400_**	<<3	<3	***	***
**Fibrin_800_**	~3	3–7	0.16	4.33
**Fibrin_800-400_**	~6	6–10	0.14	4.86

Note: * µ_max_ = maximum proliferation rate determined in the exponential phase; ** t_d_ = doubling time calculated from the beginning of the exponential phase; *** not estimated. Out of the range of the experimental data.

## Supplementary Materials

The following are available online at https://www.mdpi.com/1420-3049/24/15/2729/s1, Figure S1: Concentration of (**A**) platelets, (**B**) leukocytes, (**C**) lymphocytes and (**D**) granulocytes in L-PRPs of the donors 2 and 3, as measured in a hematologic analyzer. The calculated ratios of (**E**) platelet/leukocyte from (**A**,**B**) and (**F**) lymphocyte/granulocyte from (**C**,**D**). Note: * *p* < 0.05. Figure S2: Kinetics of the GFs (**A**) TGF-β1, (**B**) PDGF-BB, and the inflammatory cytokines (**C**) IL-8 and (**D**) TNF-α released for 72 h from fibrin nanofibers prepared using the L-PRP of two distinct donors (donor 2 and donor 3). The profiles show the strong influence of the centrifugation condition on the soluble factors release and the reproducibility of the tendencies between the donors.
