# Supplementary material for: Centrifugation Conditions in the L-PRP Preparation Affect Soluble Factors Release and Mesenchymal Stem Cell Proliferation in Fibrin Nanofibers
Source: Molecules. 2019 Jul 27;24(15):2729. doi: 10.3390/molecules24152729 (PMC6696255; doi:10.3390/molecules24152729)
Supplement: Supplementary file 1 [file molecules-24-02729-s001.pdf]

*Supplementary Material*

# Centrifugation Conditions in the L-PRP Preparation Affect Soluble Factors Release and Mesenchymal Stem Cells Proliferation in Fibrin Nanofibers

Bruna Alice Gomes de Melo, Ângela Cristina Malheiros Luzo, José Fabio Santos Duarte Lana & Maria Helena Andrade Santana

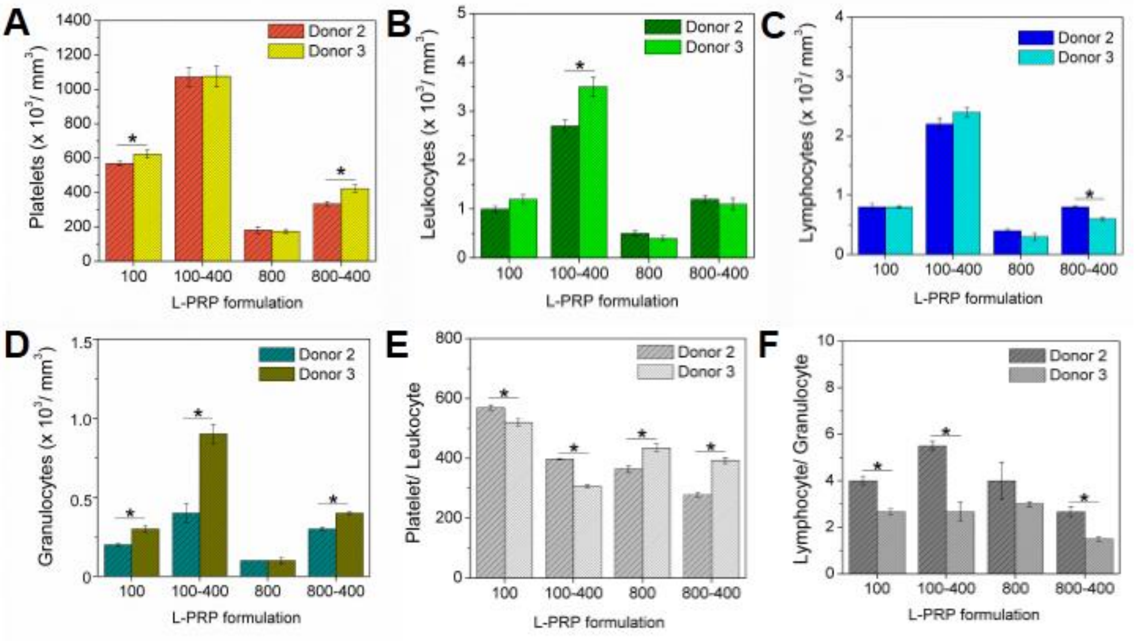


**Figure S1.** Concentration of (**A**) platelets, (**B**) leukocytes, (**C**) lymphocytes and (**D**) granulocytes in L-PRPs of the donors 2 and 3, as measured in a hematologic analyzer. The calculated ratios of (E) platelet/leukocyte from (**A**,**B**) and (**F**) lymphocyte/granulocyte from (**C**,**D**). Note: *$p < 0.05$.

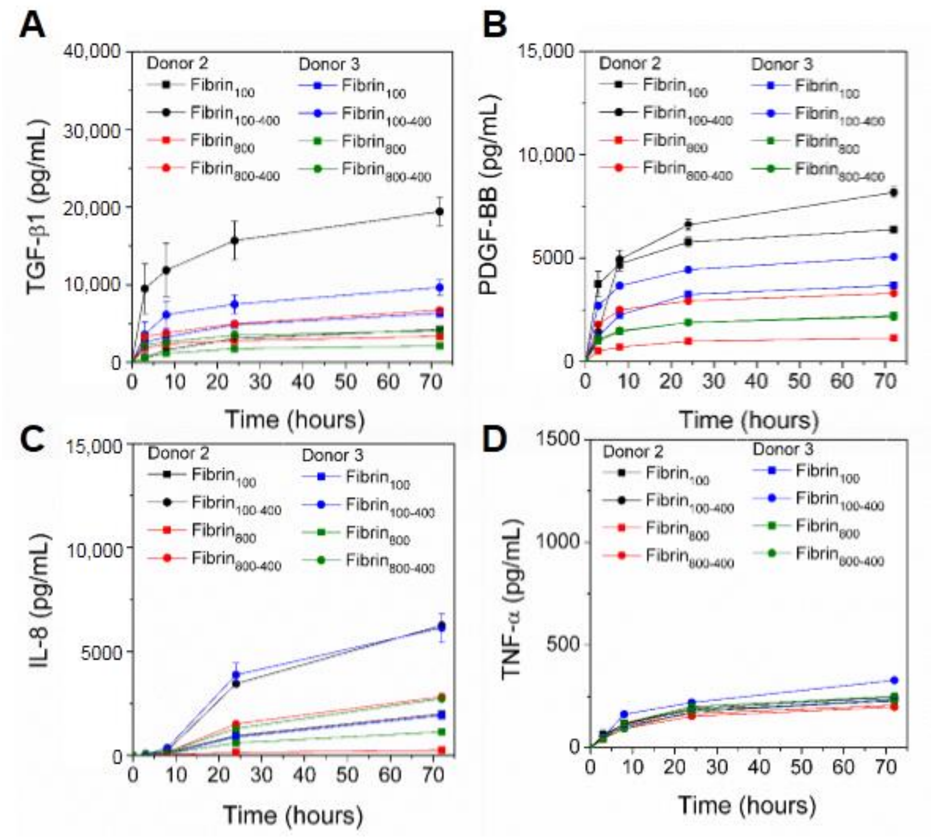


**Figure S2.** Kinetics of the GFs (**A**) TGF-β1, (**B**) PDGF-BB, and the inflammatory cytokines (**C**) IL-8 and (**D**) TNF-$\alpha$ released for 72 h from fibrin nanofibers prepared using the L-PRP of two distinct donors (donor 2 and donor 3). The profiles show the strong influence of the centrifugation condition on the soluble factors release and the reproducibility of the tendencies between the donors.
